# 1-(3,4-Dimethyl­benzyl­idene)-4-ethyl­thio­semicarbazide

**DOI:** 10.1107/S1600536810038389

**Published:** 2010-09-30

**Authors:** Yu-Feng Li, Fan-Yong Meng

**Affiliations:** a Microscale Science Institute, Department of Chemistry and Chemical Engineering, Weifang University, Weifang 261061, People’s Republic of China; b Weifang Middle School, Weifang 261061, People’s Republic of China

## Abstract

The title compound, C_12_H_17_N_3_S, was prepared by the reaction of 4-ethyl­thio­semicarbazide and 3,4-dimethyl­benzaldehyde. The dihedral angle between the thiourea unit and the benzene ring is 7.09 (8)°. In the crystal, inversion dimers linked by pairs of N—H⋯S hydrogen bonds occur.

## Related literature

For applications of Schiff base compounds, see: Casas *et al.* (2000 ▶); Habermehl *et al.* (2006 ▶). For the structure of 4-ethyl-1-(4-methyl­benzyl­idene)thio­semicarbazide, see: Li & Jian (2010 ▶).
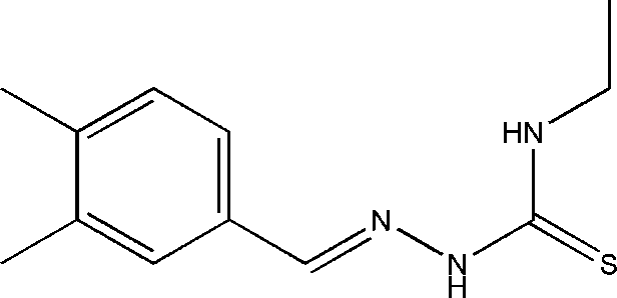


## Experimental

### 

#### Crystal data

C_12_H_17_N_3_S
*M* *_r_* = 235.35
Monoclinic, 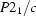

*a* = 8.6659 (17) Å
*b* = 15.207 (3) Å
*c* = 9.993 (2) Å
β = 93.47 (3)°
*V* = 1314.5 (5) Å^3^
*Z* = 4
Mo *K*α radiation
μ = 0.23 mm^−1^
*T* = 293 K
0.22 × 0.20 × 0.18 mm

#### Data collection

Bruker SMART CCD diffractometer
12215 measured reflections
3006 independent reflections
2429 reflections with *I* > 2σ(*I*)
*R*_int_ = 0.056

#### Refinement

*R*[*F* ^2^ > 2σ(*F* ^2^)] = 0.065
*wR*(*F* ^2^) = 0.206
*S* = 1.05
3006 reflections
145 parameters
H-atom parameters constrained
Δρ_max_ = 0.39 e Å^−3^
Δρ_min_ = −0.34 e Å^−3^

### 

Data collection: *SMART* (Bruker, 1997 ▶); cell refinement: *SAINT* (Bruker, 1997 ▶); data reduction: *SAINT*; program(s) used to solve structure: *SHELXS97* (Sheldrick, 2008 ▶); program(s) used to refine structure: *SHELXL97* (Sheldrick, 2008 ▶); molecular graphics: *SHELXTL* (Sheldrick, 2008 ▶); software used to prepare material for publication: *SHELXTL*.

## Supplementary Material

Crystal structure: contains datablocks global, I. DOI: 10.1107/S1600536810038389/lh5136sup1.cif

Structure factors: contains datablocks I. DOI: 10.1107/S1600536810038389/lh5136Isup2.hkl

Additional supplementary materials:  crystallographic information; 3D view; checkCIF report

## Figures and Tables

**Table 1:** Hydrogen-bond geometry (Å, °)

*D*—H⋯*A*	*D*—H	H⋯*A*	*D*⋯*A*	*D*—H⋯*A*
N2—H2*A*⋯S1^i^	0.86	2.65	3.4929 (18)	168

Symmetry code: (i) 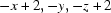.

**Fig. 1.**
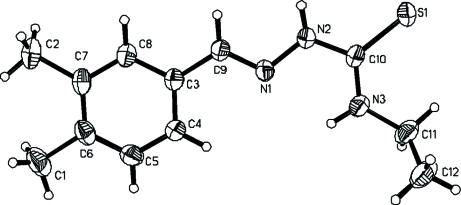
The molecular structure of the title compound showing 30% probability displacement ellipsoids and the atom-numbering scheme.

## supplementary crystallographic information

### Comment

Schiff-bases have attracted attention because they can be utilized as effective ligands in coordination chemistry (Casas *et al.*, 2000). They are important intermediates which have been reported to form chiral coordination compounds with many interesting properties (Habermehl *et al.*, 2006). As part of our research on new Schiff-base compounds we synthesized the title compound (I), and have determined its crystal structure. The molecular structure is shown in Fig. 1. The dihedral angle between the benzene ring and the thiourea unit is 7.09 (8)°. The bond lengths and angles agree with those observed in 4-Ethyl-1-(4-methylbenzylidene)thiosemicarbazide (Li & Jian, 2010). In the crystal structure, centrosymmetric dimers are formed by pairs of intermolecular N—H···S hydrogen bonds.

### Experimental

A mixture of the 4-ethylthiosemicarbazide (0.1 mol) and 3,4-dimethylbenzaldehyde (0.1 mol) was stirred in refluxing ethanol (30 mL) for 2 h to afford the title compound (0.085 mol, yield 85%). Single crystals suitable for X-ray measurements were obtained by recrystallization of a solution of the title compound in ethanol at room temperature.

### Refinement

H atoms were fixed geometrically and allowed to ride on their attached atoms, with C—H distances = 0.93-0.97 Å; N-H = 0.86Å, and with *U*_iso_ = 1.2*U*_eq_(C,N) or 1.2*U*_eq_(C_methyl_).

### Crystal data

**Table:**

C_12_H_17_N_3_S	*F*(000) = 504
*M**_r_* = 235.35	*D*_x_ = 1.189 Mg m^−^^3^
Monoclinic, *P*2_1_/*c*	Mo *K*α radiation, λ = 0.71073 Å
Hall symbol: -P 2ybc	Cell parameters from 2429 reflections
*a* = 8.6659 (17) Å	θ = 3.3–27.5°
*b* = 15.207 (3) Å	µ = 0.23 mm^−^^1^
*c* = 9.993 (2) Å	*T* = 293 K
β = 93.47 (3)°	Block, colorless
*V* = 1314.5 (5) Å^3^	0.22 × 0.20 × 0.18 mm
*Z* = 4	

### Data collection

**Table:**

Bruker SMART CCD diffractometer	2429 reflections with *I* > 2σ(*I*)
Radiation source: fine-focus sealed tube	*R*_int_ = 0.056
graphite	θ_max_ = 27.5°, θ_min_ = 3.3°
φ and ω scans	*h* = −11→11
12215 measured reflections	*k* = −19→19
3006 independent reflections	*l* = −12→11

### Refinement

**Table:**

Refinement on *F*^2^	Primary atom site location: structure-invariant direct methods
Least-squares matrix: full	Secondary atom site location: difference Fourier map
*R*[*F*^2^ > 2σ(*F*^2^)] = 0.065	Hydrogen site location: inferred from neighbouring sites
*wR*(*F*^2^) = 0.206	H-atom parameters constrained
*S* = 1.05	*w* = 1/[σ^2^(*F*_o_^2^) + (0.1293*P*)^2^ + 0.2681*P*] where *P* = (*F*_o_^2^ + 2*F*_c_^2^)/3
3006 reflections	(Δ/σ)_max_ < 0.001
145 parameters	Δρ_max_ = 0.39 e Å^−^^3^
0 restraints	Δρ_min_ = −0.34 e Å^−^^3^

### Special details

**Table:**

Geometry. All e.s.d.'s (except the e.s.d. in the dihedral angle between two l.s. planes) are estimated using the full covariance matrix. The cell e.s.d.'s are taken into account individually in the estimation of e.s.d.'s in distances, angles and torsion angles; correlations between e.s.d.'s in cell parameters are only used when they are defined by crystal symmetry. An approximate (isotropic) treatment of cell e.s.d.'s is used for estimating e.s.d.'s involving l.s. planes.
Refinement. Refinement of *F*^2^ against ALL reflections. The weighted *R*-factor *wR* and goodness of fit *S* are based on *F*^2^, conventional *R*-factors *R* are based on *F*, with *F* set to zero for negative *F*^2^. The threshold expression of *F*^2^ > σ(*F*^2^) is used only for calculating *R*-factors(gt) *etc*. and is not relevant to the choice of reflections for refinement. *R*-factors based on *F*^2^ are statistically about twice as large as those based on *F*, and *R*- factors based on ALL data will be even larger.

### Fractional atomic coordinates and isotropic or equivalent isotropic displacement parameters (Å^2^)

**Table:**

	*x*	*y*	*z*	*U*_iso_*/*U*_eq_	
S1	0.84996 (8)	0.11266 (4)	0.92188 (6)	0.0702 (3)	
N1	1.02915 (18)	0.11306 (10)	1.28935 (16)	0.0480 (4)	
N2	0.9879 (2)	0.08927 (11)	1.15935 (16)	0.0532 (4)	
H2A	1.0235	0.0415	1.1269	0.064*	
C9	1.1228 (2)	0.06157 (13)	1.35388 (19)	0.0513 (4)	
H9A	1.1574	0.0115	1.3114	0.062*	
N3	0.8377 (2)	0.21192 (12)	1.14109 (18)	0.0581 (4)	
H3A	0.8630	0.2193	1.2249	0.070*	
C4	1.1328 (3)	0.15253 (16)	1.5627 (2)	0.0597 (5)	
H4A	1.0691	0.1947	1.5206	0.072*	
C8	1.2782 (2)	0.01920 (13)	1.5576 (2)	0.0544 (5)	
H8A	1.3120	−0.0293	1.5108	0.065*	
C3	1.1773 (2)	0.07867 (13)	1.49181 (19)	0.0487 (4)	
C10	0.8916 (2)	0.14075 (13)	1.08270 (19)	0.0485 (4)	
C7	1.3299 (2)	0.03031 (14)	1.6911 (2)	0.0571 (5)	
C6	1.2793 (3)	0.10241 (17)	1.7607 (2)	0.0644 (6)	
C5	1.1829 (3)	0.16286 (18)	1.6946 (2)	0.0708 (6)	
H5A	1.1511	0.2120	1.7409	0.085*	
C2	1.4415 (3)	−0.0353 (2)	1.7562 (3)	0.0837 (8)	
H2B	1.4651	−0.0188	1.8480	0.126*	
H2C	1.5348	−0.0360	1.7092	0.126*	
H2D	1.3956	−0.0927	1.7530	0.126*	
C12	0.5844 (3)	0.2822 (2)	1.1272 (4)	0.0950 (9)	
H12A	0.5239	0.3265	1.0800	0.143*	
H12B	0.5935	0.2963	1.2210	0.143*	
H12C	0.5348	0.2261	1.1149	0.143*	
C11	0.7391 (3)	0.27866 (18)	1.0749 (3)	0.0787 (7)	
H11A	0.7289	0.2663	0.9796	0.094*	
H11B	0.7881	0.3357	1.0868	0.094*	
C1	1.3292 (4)	0.1167 (3)	1.9069 (3)	0.0973 (11)	
H1B	1.2832	0.1698	1.9380	0.146*	
H1C	1.4397	0.1216	1.9166	0.146*	
H1D	1.2962	0.0679	1.9589	0.146*	

### Atomic displacement parameters (Å^2^)

**Table:**

	*U*^11^	*U*^22^	*U*^33^	*U*^12^	*U*^13^	*U*^23^
S1	0.0834 (5)	0.0734 (4)	0.0509 (4)	0.0196 (3)	−0.0188 (3)	−0.0005 (2)
N1	0.0472 (8)	0.0524 (8)	0.0437 (8)	0.0018 (6)	−0.0027 (6)	0.0016 (6)
N2	0.0575 (9)	0.0541 (8)	0.0464 (8)	0.0123 (7)	−0.0088 (7)	−0.0005 (7)
C9	0.0536 (10)	0.0496 (9)	0.0496 (10)	0.0050 (7)	−0.0049 (8)	0.0006 (8)
N3	0.0575 (9)	0.0611 (10)	0.0554 (9)	0.0163 (8)	0.0018 (7)	0.0074 (7)
C4	0.0582 (11)	0.0668 (13)	0.0535 (11)	0.0141 (9)	−0.0015 (8)	−0.0024 (9)
C8	0.0552 (10)	0.0493 (9)	0.0572 (11)	−0.0028 (8)	−0.0075 (8)	0.0059 (8)
C3	0.0461 (9)	0.0515 (9)	0.0481 (9)	−0.0020 (7)	−0.0019 (7)	0.0045 (8)
C10	0.0414 (9)	0.0526 (9)	0.0510 (10)	0.0036 (7)	−0.0019 (7)	0.0086 (8)
C7	0.0505 (10)	0.0641 (11)	0.0554 (11)	−0.0114 (8)	−0.0088 (8)	0.0156 (9)
C6	0.0523 (11)	0.0943 (16)	0.0459 (11)	−0.0092 (10)	−0.0025 (8)	0.0010 (10)
C5	0.0677 (14)	0.0861 (16)	0.0580 (12)	0.0112 (11)	0.0003 (10)	−0.0162 (11)
C2	0.0802 (16)	0.0911 (18)	0.0768 (16)	0.0046 (13)	−0.0208 (13)	0.0262 (14)
C12	0.0636 (15)	0.0908 (19)	0.128 (3)	0.0270 (14)	−0.0117 (15)	−0.0062 (18)
C11	0.0817 (16)	0.0739 (15)	0.0810 (16)	0.0347 (13)	0.0081 (12)	0.0185 (12)
C1	0.089 (2)	0.150 (3)	0.0508 (14)	−0.0023 (18)	−0.0132 (13)	−0.0072 (15)

### Geometric parameters (Å, °)

**Table:**

S1—C10	1.681 (2)	C7—C2	1.509 (3)
N1—C9	1.275 (2)	C6—C5	1.383 (3)
N1—N2	1.375 (2)	C6—C1	1.514 (3)
N2—C10	1.349 (2)	C5—H5A	0.9300
N2—H2A	0.8600	C2—H2B	0.9600
C9—C3	1.453 (3)	C2—H2C	0.9600
C9—H9A	0.9300	C2—H2D	0.9600
N3—C10	1.328 (3)	C12—C11	1.468 (4)
N3—C11	1.459 (3)	C12—H12A	0.9600
N3—H3A	0.8600	C12—H12B	0.9600
C4—C5	1.371 (3)	C12—H12C	0.9600
C4—C3	1.395 (3)	C11—H11A	0.9700
C4—H4A	0.9300	C11—H11B	0.9700
C8—C7	1.392 (3)	C1—H1B	0.9600
C8—C3	1.395 (3)	C1—H1C	0.9600
C8—H8A	0.9300	C1—H1D	0.9600
C7—C6	1.384 (3)		
			
C9—N1—N2	115.95 (16)	C4—C5—C6	122.0 (2)
C10—N2—N1	120.03 (16)	C4—C5—H5A	119.0
C10—N2—H2A	120.0	C6—C5—H5A	119.0
N1—N2—H2A	120.0	C7—C2—H2B	109.5
N1—C9—C3	122.03 (18)	C7—C2—H2C	109.5
N1—C9—H9A	119.0	H2B—C2—H2C	109.5
C3—C9—H9A	119.0	C7—C2—H2D	109.5
C10—N3—C11	125.4 (2)	H2B—C2—H2D	109.5
C10—N3—H3A	117.3	H2C—C2—H2D	109.5
C11—N3—H3A	117.3	C11—C12—H12A	109.5
C5—C4—C3	119.9 (2)	C11—C12—H12B	109.5
C5—C4—H4A	120.0	H12A—C12—H12B	109.5
C3—C4—H4A	120.0	C11—C12—H12C	109.5
C7—C8—C3	121.9 (2)	H12A—C12—H12C	109.5
C7—C8—H8A	119.0	H12B—C12—H12C	109.5
C3—C8—H8A	119.0	N3—C11—C12	112.7 (2)
C8—C3—C4	117.90 (18)	N3—C11—H11A	109.0
C8—C3—C9	119.30 (18)	C12—C11—H11A	109.0
C4—C3—C9	122.80 (18)	N3—C11—H11B	109.0
N3—C10—N2	116.46 (17)	C12—C11—H11B	109.0
N3—C10—S1	124.51 (14)	H11A—C11—H11B	107.8
N2—C10—S1	119.02 (15)	C6—C1—H1B	109.5
C6—C7—C8	119.00 (19)	C6—C1—H1C	109.5
C6—C7—C2	121.4 (2)	H1B—C1—H1C	109.5
C8—C7—C2	119.6 (2)	C6—C1—H1D	109.5
C5—C6—C7	119.1 (2)	H1B—C1—H1D	109.5
C5—C6—C1	119.6 (2)	H1C—C1—H1D	109.5
C7—C6—C1	121.2 (2)		

### Hydrogen-bond geometry (Å, °)

**Table:**

*D*—H···*A*	*D*—H	H···*A*	*D*···*A*	*D*—H···*A*
N2—H2A···S1^i^	0.86	2.65	3.4929 (18)	168

Symmetry codes: (i) −*x*+2, −*y*, −*z*+2.
